# The CARAMAL study could not assess the effectiveness of rectal artesunate in treating suspected severe malaria

**DOI:** 10.1186/s12916-023-02776-z

**Published:** 2023-03-30

**Authors:** James A. Watson, Thomas J. Peto, Nicholas J. White

**Affiliations:** 1 Oxford University Clinical Research Unit, Hospital for Tropical Diseases, Ho Chi Minh City, Viet Nam; 2 Centre for Tropical Medicine and Global Health, Nuffield Department of Medicine, University of Oxford, Oxford, UK; 3 Mahidol Oxford Tropical Medicine Research Unit, Faculty of Tropical Medicine, Mahidol University, Bangkok, Thailand

**Keywords:** Rectal artesunate, Severe malaria, World Health Organization

## Abstract

CARAMAL was a large observational study which recorded mortality in children with suspected severe malaria before and after the roll-out of rectal artesunate in Nigeria, Uganda and the Democratic Republic of the Congo. The results of CARAMAL have had a huge impact on public health policy leading to a World Health Organization moratorium on the roll-out of rectal artesunate. The conclusion reported in the abstract uses strong causal language, stating that “pre-referral RAS [rectal artesunate suppositories] had no beneficial effect on child survival”. We argue that this causal interpretation of the study results is not justified. Data from the CARAMAL study inform chiefly on the strengths and weaknesses of referral systems in these three countries and do not inform reliably as to the beneficial effect of providing access to a known life-saving treatment.

## Background

Severe malaria is a medical emergency for which artesunate is the best available life-saving treatment. Primary care in most rural malaria-endemic parts of sub-Saharan Africa is provided by small health clinics or by community health workers who cannot administer parenteral treatment. Thus, rapid recognition and referral to a hospital is critical for the appropriate treatment and management of potentially lethal infections. Delays in reaching the hospital have fatal consequences. In the pre-referral treatment of suspected severe malaria, rectal artesunate suppositories (RAS) are safe, can be administered by community health workers, and provide therapeutic concentrations in blood which reduce malaria parasite burdens by a factor of approximately 10,000 within 48 h [1]. RAS buys critical extra time for a child with severe malaria whilst they are being referred. The robust evidence from RCTs that parenteral artesunate is the best available treatment for severe malaria [2, 3] and the evidence on rectal bioavailability [4] together provide a very strong therapeutic rationale for RAS. For community-administered severe malaria interventions, the difficulty in distinguishing severe malaria from other life-threatening illnesses considerably dilutes mortality effect sizes. The largest double-blind randomised trial (Study 13) of RAS [5] did not meet statistical significance when assessed in all randomised patients, but suggested a substantial benefit in those children with severe falciparum malaria presenting after 6 h to the hospital (post hoc subgroup analysis). Development and deployment of RAS have gone very slowly, and now, as a direct result of the CARAMAL study, their deployment has been halted [6–8].

The CARAMAL study was an observational study in Nigeria, Uganda and the Democratic Republic of the Congo (DRC) which recorded patient outcomes before and after the roll-out of RAS and, after roll-out, recorded patient outcomes in those children who did and did not receive RAS [9]. The CARAMAL study provides important data on the performance of primary care and the referral processes in these three countries, notably highlighting low referral completion rates. However, increases in mortality over time in this pre- versus post-observational study have been linked *causally* to RAS roll-out. The CARAMAL study reported considerably higher mortality after the RAS roll-out in Nigeria and higher mortality in children receiving RAS compared to those not receiving RAS (in DRC and in Nigeria, where the difference was marked). There is no evidence for population differences in the pharmacokinetics or antimalarial pharmacodynamics of artesunate, but there are differences in prescribing, behavior, ascertainment, referral patterns and health care availability. These factors affect therapeutic outcomes for life-threatening infections and can have a strong influence on observational data. These factors may have contributed to differences between the pre- versus post-observational CARAMAL study and the earlier double-blind RCT.

The conclusion of the Hetzel et al. publication is “pre-referral RAS had no beneficial effect on child survival” [9]. This is an unambiguous causal interpretation given to observational data which we strongly believe to be incorrect. This conclusion from an observational study led directly to a World Health Organization (WHO) moratorium on RAS roll-out in Africa (before the appearance of any of the publications in peer-reviewed journals)—an extremely serious decision with far-reaching consequences. We argue that these observational data cannot be interpreted causally and cannot provide estimates of the “effectiveness” of RAS. Major concerns have been published previously based on the pre-print versions of the study results [10]. Here, we focus specifically on the causal language used in the conclusion of the abstract in the now published report and on technical concerns with the analysis and reporting [9].

## Two sources of confounding bias

### COVID-19 and community engagement could have changed the patient population over time

One of the primary research questions of the CARAMAL study was “Can the introduction of pre-referral QA RAS [quality-assured rectal artesunate] reduce the severe malaria case fatality ratio over time under real-world operational circumstances in three distinct settings?” (see clinicaltrials.gov: NCT03568344). Describing this in simpler terms, the CARAMAL study aimed to assess the *effectiveness* of RAS (i.e. population-level impact of RAS) as opposed to the *efficacy* of RAS (individual-level effect when administered correctly). Whereas individually randomised trials typically estimate efficacy, cluster randomised or stepped-wedge randomised trials (and, in theory, well-designed observational studies) can provide effectiveness estimates. Effectiveness encompasses how health workers use and prescribe the treatment and what happens to patients after treatment is received (in this case, whether they are referred rapidly to a larger health care facility, whether and when they are given parenteral artesunate, and whether this is followed by a complete course of an artemisinin-based combination therapy).

**Fig. 1:**
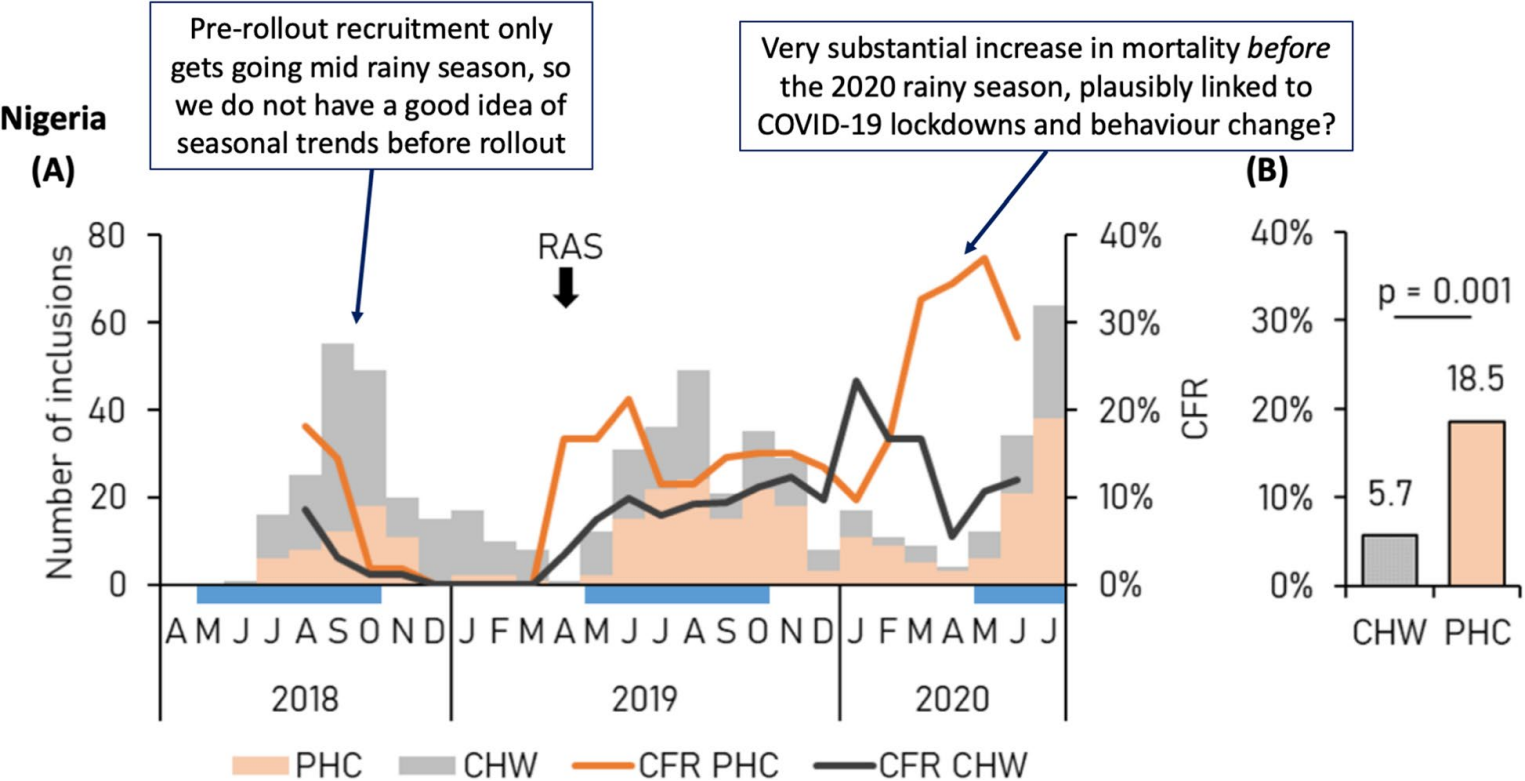
Mortality trends over time as shown in Fig. S5 of Hetzel et al [9]. CHW, community health worker; PHC, primary health center; CFR, case fatality ratio; RAS, rectal artesunate suppository

With this objective, the CARAMAL study compared mortality in children presenting to primary healthcare clinics or village health care workers before versus after the RAS roll-out. The most notable result was a 4-fold increase in mortality after the RAS roll-out in Nigeria (16.1% versus 4.2%, $p<0.001$). No statistically significant differences in post- versus pre-roll-out mortality were observed in the other two countries. Interpreting mortality increases after RAS roll-out as being *caused* by RAS itself is highly problematic because that interpretation assumes all the other key determinants of mortality had remained the same. The pre-roll-out period in Nigeria covered only the latter half of the rainy season in 2018 (peak recruitment occurred in the last 2 months of the rainy season, September and October, Fig. S5 reproduced in Fig. 1). Over two-thirds of the enrollment in this period was done by community health workers. Mortality trends during the 2019 rainy season (post-roll-out) appear very similar although over half the patients in the latter period were recruited at primary health centres, not by community health workers as in the pre-roll-out phase. The largest increase in mortality occurred *before* the 2020 rainy season (February to May 2020), during which health-seeking behaviour could have changed substantially as a result of the COVID-19 pandemic. We do not know the extent to which COVID-19 disrupted health services and health-seeking behavior in the study areas but it seems likely the disruption was substantial. Because Fig. S5 shows a 3-month moving average mortality, we cannot derive from the graph the exact timing of the increase in deaths and how this relates to COVID-19 pandemic related behavioural changes, but it appears that the majority of these excess deaths were not from malaria. The authors do report a sensitivity analysis excluding the post-COVID-19 follow-up data, but this was done only for the RAS use versus no RAS use analysis and not for the pre- versus post-roll-out analysis (see next section). Thus, the following statement in the Discussion does not seem justified: “while COVID-19 pandemic measures may have influenced treatment seeking or provision of care, limiting the health outcome analyses to the pre-Covid-19 period did not change the observed effect of RAS”.

The claim that the increase in mortality in Nigeria is *causally* related to the RAS roll-out itself depends on the assumption that all other major determinants of health care seeking and outcome were held equal: i.e. that the group of patients recruited and the ascertainment before the RAS roll-out were comparable with the group of patients recruited after roll-out. The COVID-19 epidemic and its disastrous effect on health care in low- and middle-income countries around the world makes this assumption improbable.

Another reason why the pre- and post-roll-out populations were different is that the CARAMAL study itself aimed explicitly to change health care-seeking behavior. As stated in the end-of-grant report: “The CARAMAL project worked closely with the Ministries of Health to design context-specific strategies with a goal to improve care-seeking behavior among community members, promote the use of RAS among CHWs [community health workers] and encourage completion of treatment among caregivers” [11]. Quantifying the effect of these engagement strategies on health care-seeking behaviour is extremely difficult, but this aspect of the study could have plausibly had important effects in the villages where CARAMAL was carried out. Could the parent of a very sick child after the RAS roll-out have decided to consult the health worker because they knew that a potentially life-saving treatment had become available? Before roll-out, that child might never have presented to a primary care facility and instead have been brought directly to a larger hospital and died en route, or not have been referred to medical attention at all and died uncounted at home? We cannot know or characterise the exact biases at play here, but it does not seem justified to claim pre- and post-roll-out periods were comparable.

### Was RAS given to the sicker children (confounding by indication)?

The second major finding reported in the abstract is that “in DRC and Nigeria, children receiving RAS were more likely to die than those not receiving RAS (aOR=3.06, 95% CI 1.35-6.92 and aOR=2.16, 95% CI 1.11-4.21, respectively)”. These adjusted odds ratios are very large. In Nigeria, the mortality was 19.7% in patients who received RAS versus 7.7% in patients who did not receive RAS. The majority of these excess deaths in the RAS group occurred within 48 h of enrolment (9% versus 4%). A doubling of mortality within 48 h of administration of a highly effective antimalarial drug is simply not compatible with our current understanding of the disease processes in severe falciparum malaria [12] and the pharmacokinetic and pharmacodynamic properties of RAS. It is difficult to understand how the administration of a drug which reduces the mortality of untreated severe malaria by more than 90% could result in a doubling of mortality within 48 h (i.e. one asexual parasite life cycle)? This surprising result naturally raises questions about the quality of the information. The simplest explanation for this very large difference in mortality is that health workers prioritised administering RAS to the sickest children (confounding by indication). This is especially plausible in Nigeria where there were distribution issues and only half the enrolled patients received RAS post-roll-out. It is not possible to quantify the extent of this confounding bias using the data collected during the study (e.g. by computing propensity scores): the only measure of severity at enrollment was the presence of convulsions. This binary variable cannot be expected to explain all differences in severity between the two groups. Selective administration of RAS to the sickest children (with any infection) seems the simplest explanation for this very large reported difference in mortality in patients given RAS.

### Was RAS administration reliably recorded by health workers?

We are concerned about the accuracy of the individual patient data on RAS administration. There were apparently two sources of information on the administration of RAS: the recall of the caregivers documented over 1 month after the death of the child and the documentation of the health worker who enrolled the patient. The tragedy of a child dying after having been diagnosed with severe malaria, but not being referred to a hospital for definitive treatment, must have affected adversely both the families and the health workers. The authors (and the WHO) suggested that RAS administration could have fatally reassured the caregivers, who were then less likely to take the child to the hospital [7]. But can we be sure that in children who died, the records on RAS administration were accurate? Failing to administer a potentially life-saving medicine to a child who died soon afterwards would have reflected very badly on the health worker. It is unclear how discrepancies between the two sources of information were resolved and whether any biases could have resulted.

### Why causal inference was not possible from the outset

Epidemiological studies analysing the relationship between a particular exposure of interest (e.g. RAS administration) and health outcomes (e.g. death) can only hope to provide reliable causal estimates when (i) the mechanisms that determine the exposure are both understood and recorded reliably (treatment assignment mechanisms) and (ii) when the compared groups are known to be largely similar in their characteristics. Both of these are provided by “the magic of randomisation” [13]. Neither was the case in the sequential observational CARAMAL study. CARAMAL was designed so that the data reflect “real-world observations” with minimal data collection and surveillance. The CARAMAL data are thus valuable for understanding the strengths and weaknesses of the health systems in these three countries. But the idea that these “real-world data” can provide information on the effectiveness of RAS is truly a myth [13]. In a follow-up viewpoint, the authors reinforce this misunderstanding: “controlled trials provide much less informative evidence of real-world effectiveness than observational studies do” [7]. This is fundamentally wrong.

It is well established that observational studies, even those that are large and well conducted, can yield misleading results which are opposite to those from large and definitive randomised controlled trials [13]. We cannot know from the available data why some children received RAS and why others did not. We cannot know from the available data how the enrolled patient population changed over time. We therefore cannot make statements about the *causal* effect of RAS administration on mortality after the RAS roll-out based on these data.

The authors acknowledge some of these issues in the limitations paragraph of the Discussion: “The increased CFR [case-fatality ratio] associated with the roll-out and use of RAS observed in DRC and Nigeria is likely a result of complex interactions between disease severity, treatment seeking, and care provided in the context of weak health systems, rather than a direct result of RAS treatment which was previously shown to be safe and efficacious. Secular trends in disease incidence and severity may have played a confounding role in DRC, where a larger number of severe cases were enrolled in the post-RAS period”. This complex disclaimer cannot be reconciled with the stark conclusion in the abstract: “pre-referral RAS had no beneficial effect on child survival”. This is a clear statement of cause and effect. The causal language used in the abstract, which underpins the rationale for the WHO moratorium, is not justified.

## Technical problems with the analysis

### No study protocol or statistical analysis plan

It is difficult for external researchers to evaluate properly and fairly the conduct and analysis of the CARAMAL study. No study protocol was published with the main paper. The authors of the CARAMAL study have refused to share the study protocol with us. There does not appear to have been a statistical analysis plan prepared before data analysis. Indeed, many of the analyses presented in Hetzel et al. do not align with the primary aim of the study. We understand that the primary aim was to characterise changes in mortality before and after RAS deployment (the sample size calculation was done on a pre- versus post-roll-out comparison). However, much of the analysis focuses on differences in mortality and referral between patients given RAS and those not given RAS, post-deployment. Surely this is exactly what a randomised trial should evaluate? There appears to be a logical inconsistency between the stated aims of the study and the data analysis and its interpretation.

### Incorrect adjustment for variables on the causal pathway

For the DRC, the adjusted odds ratio for death in children receiving RAS versus those not receiving RAS is reported in the abstract as 3.06 (details of the analysis are given in Table 4 [9]). Three analyses are presented in Table 4 of Hetzel et al: (i) unadjusted; (ii) adjusted for baseline covariates (sex, age $<1$ year, beginning of RAS roll-out [it is unclear what this means], convulsions, enrolment location [community health worker versus primary healthcare centre], and rainy season); and (iii) adjusted further for referral and post-referral treatment at the hospital. We note that none of these analyses was pre-specified (no statistical analysis plan is provided), and it is unclear why the analysis of RAS use was adjusted for these covariates, whereas the pre- versus post-roll-out analysis was not adjusted for any covariates (which we understand to be the primary analysis of the study). However, regardless of how these analyses were chosen, there is a fundamental error which invalidates the main reported analysis. The odds ratio of 3.06 reported in the abstract is taken from the third set of analyses (adjusted for referral and post-referral treatment). Referral (or treatment at a referral hospital) and death are competing events. In addition, referral is on the causal pathway between RAS administration and death at 28 days. A child who dies on their way to the hospital does not count as “referred” and will not be treated. It is incorrect to adjust for referral and treatment as if they were baseline variables. This is sometimes referred to as overadjustment bias [14, 15].

## Conclusions

The deployment of RAS in Africa was effectively halted by the WHO Global Malaria Programme in January 2022 [6]. This major policy change affecting child survival was a rapid response to the unpublished findings of the CARAMAL observational study. Unusually, this important policy recommendation did not undergo the usual rigorous review process that should underpin therapeutic recommendations from the WHO. The WHO GMP information note states: “the study [CARAMAL] documents the potential for harm and increased mortality if pre-referral RAS is not strictly implemented in line with existing WHO guidelines” [6]. As in the publication by Hetzel et al., this interprets observed increases in mortality as *causally* related to RAS deployment. This interpretation is likely to be wrong for the reasons we have outlined above.

We believe that much of the confusion stems from the initial design of the CARAMAL study. CARAMAL had the stated objective of characterising the causal effect of RAS deployment on childhood mortality. This causal goal for an observational study with no possibility of controlling exogenous confounding factors is misguided. Regardless of whether mortality went down, stayed the same or went up, we cannot reliably interpret temporal changes as causally linked with RAS deployment. This overconfidence in the value of observational data is highlighted in the discussion of Hetzel et al., which states: “The current recommendation to use RAS as pre-referral treatment where parenteral alternatives are unavailable is based on a randomised controlled trial that provided little evidence of the effect of introducing RAS at scale” [9]. This is incorrect and it is misleading. First, most interventions are provided at scale without implementation studies. Second, the use of RAS as pre-referral treatment is not only supported by a large multi-centre randomised trial [5], but also by (i) a strong mechanistic understanding of the disease [12], (ii) detailed pharmacometric studies showing adequate drug absorption and rapid parasite clearance [1, 4], (iii) an excellent safety profile [16], and (iv) large randomised controlled trials which show unequivocally the substantial superiority of artesunate in the treatment of severe malaria [2, 3].

Epidemiological studies with causal goals have utility in contexts where either (i) there is a clear natural experiment which provides reassurance that unmeasured confounding will have a minimal effect (e.g. [17]) or (ii) where effect sizes are so large that moderate biases have a minimal influence on effect estimates [13]. RAS deployment in malaria-endemic areas of sub-Saharan Africa is not expected to half childhood mortality. A large proportion of patients with malaria-positive RDTs presenting to primary health centres or to community health workers will not have severe malaria [18]. RAS will save lives in the sub-population of children with true severe malaria, but its effect on overall mortality in the enrolled population will be moderate (the sample size calculation for CARAMAL posited a 30% reduction in mortality post-RAS roll-out). As the incidence of malaria falls, the overall effect on mortality will decline as malaria comprises a progressively smaller proportion of severe childhood illnesses. Observational studies in general cannot distinguish reliably between moderate effects and moderate biases. Observational research is useful to understand obstacles and optimise deployment, but randomised trial data and mechanistic reasoning are the most reliable guides in estimating treatment effects and informing RAS deployment at scale. The CARAMAL study could not assess the effectiveness of rectal artesunate in treating suspected severe malaria.

## Data Availability

Not applicable.

## Abbreviations

RAS: Rectal artesunate suppositories
DRC: Democratic Republic of the Congo
WHO: World Health Organization
QA RAS: Quality-assured rectal artesunate
CHW: Community health worker
PHC: Primary health center
CFR: Case fatality ratio
